# Design Optimization and Fabrication of a Novel Structural SOI Piezoresistive Pressure Sensor with High Accuracy

**DOI:** 10.3390/s18020439

**Published:** 2018-02-02

**Authors:** Chuang Li, Francisco Cordovilla, R. Jagdheesh, José L. Ocaña

**Affiliations:** E.T.S. Ingenieros Industriales, Polytechnical University of Madrid, C/José Gutiérrez Abascal, 2. 28006 Madrid, Spain; francisco.cordovilla.baro@upm.es (F.C.); r.jagdheesh@upm.es (R.J.); jlocana@etsii.upm.es (J.L.O.)

**Keywords:** piezoresistive pressure sensor, high sensitivity, low pressure nonlinearity error, SOI structure, micro-pressure measurement, J0101

## Abstract

This paper presents a novel structural piezoresistive pressure sensor with four-grooved membrane combined with rood beam to measure low pressure. In this investigation, the design, optimization, fabrication, and measurements of the sensor are involved. By analyzing the stress distribution and deflection of sensitive elements using finite element method, a novel structure featuring high concentrated stress profile (HCSP) and locally stiffened membrane (LSM) is built. Curve fittings of the mechanical stress and deflection based on FEM simulation results are performed to establish the relationship between mechanical performance and structure dimension. A combination of FEM and curve fitting method is carried out to determine the structural dimensions. The optimized sensor chip is fabricated on a SOI wafer by traditional MEMS bulk-micromachining and anodic bonding technology. When the applied pressure is 1 psi, the sensor achieves a sensitivity of 30.9 mV/V/psi, a pressure nonlinearity of 0.21% FSS and an accuracy of 0.30%, and thereby the contradiction between sensitivity and linearity is alleviated. In terms of size, accuracy and high temperature characteristic, the proposed sensor is a proper choice for measuring pressure of less than 1 psi.

## 1. Introduction

The phenomenon by which the electrical resistance of a material changes in response to mechanical stress is known as piezoresistivity. Piezoresistivity in semiconductor is widely applied in different sensors including pressure sensors, accelerometers, cantilever force sensors, and inertial sensors [1]. Piezoresistive pressure sensors utilize piezoresistive effect as the detection mechanism. A Wheatstone bridge is built through electric connections with four piezoresistors to transduce the resistance change into output voltage when pressure is applied on the membrane surface. Owing to their small scale, easy integration, direct signal transduction mechanism, etc. [2], piezoresistive pressure sensors have been most commonly used in automobile [3], aerospace [4,5] and petrochemical [6] fields for pressure measurements.

For many years in the past, monocrystalline silicon strain gauges have been mainly chosen as the detection elements in pressure sensors. Their main characteristics include good sensitivity, high mechanical stability and batch fabrication capability owing to the single crystal structure [7,8]. For such traditional type of sensors, the p-n junction formed by the resistor with the bulk plays a main role in isolation of the resistors from substrate. However, once the temperature exceeds 125 °C, there exists serious degradation in the performance of the sensor due to leakage currents [9]. To solve this problem, some wide band gap semiconductors such as SiC and diamond have been explored as alternatives, but their microfabrication process is not mature enough [10,11]. Polysilicon can also be a viable candidate at high temperature because of an isolating oxide layer under the polysilicon resistor. However, some undesirable characteristics, such as low sensitivity, high noise, and repeatability problems, limit its further development [12,13].

By considering the working condition, the device should maintain their functionalities even at high temperatures. SOI (silicon on insulator) stands out as the most promising candidate. The fabrication of the SOI wafer has been generically described as the bonded wafer approach. As the name implies, two wafers are bonded together by thermally grown silicon oxide (SiO_2_) [14]. This structure solves many drawbacks, such as sensibility to radiation, current leakage and instability at high temperature. Thus, SOI allows piezoresistive pressure sensors to work at an operating temperature range up to 300 °C [15,16]. Besides, SOI piezoresistive pressure sensors give the combined advantages of standard silicon technology and isolation of piezoresistors from substrate, which makes SOI one of the best recognized and promoted material for piezoresistive pressure sensors [17].

For conventional structural pressure sensors, the trade-off between sensitivity and linearity is always irreconcilable, especially in the field of low pressure measurements [18]. Since sensitivity is proportional to the ratio (membrane length)/(membrane thickness), namely (*L*/*H*). Unfortunately, the nonlinearity increases with this ratio at a faster rate, as the nonlinearity of the pressure-to-stress conversion is proportional to (*L*/*H*)^4^ [19]. However, both sensitivity and linearity are important performance indicators, which may directly determine consumer satisfaction degree and market share. To achieve a higher sensitivity and alleviate the contradiction between the sensitivity and linearity simultaneously, many novel structurally sensitive membranes, such as E-type membrane [20], hollow reinforced membrane [21], circular grooved membrane [22], peninsula structural membrane [23], cross-beam membrane (CBM) [24], beam-membrane-mono-island (BMMI) [25], beam-membrane-dual-island (BMDI) [26], beam-membrane-quad-island (BMQI) [27], etc., were developed in the past decades. By comparing and analyzing such novel structures, it is found they have some common features. Either they create stress concentration regions (SCRs) and localize more strain energy within a relatively narrow space, or they locally stiffen the membrane thereby restricting partially the deformation but not affecting the stress concentration in the sensitive areas. Just because of the formation of stress concentration and locally stiffened membrane, the sensitivity and linearity both achieve a big improvement.

In this paper, a novel structural piezoresistive pressure sensor with four-grooved membrane combined with rood beam was proposed for low pressure measurement (0~1 psi) based on SOI substrate. By choosing SOI as the sensor chip, a good high temperature performance was desired. By introducing the grooves and rood beam to sensitive membrane, a high sensitivity and a low nonlinearity error were anticipated to be achieved simultaneously. To optimize the structural dimensions, the finite element method (FEM) and the nonlinear curve fitting method were implemented. Based on the optimization results, the proposed structure was fabricated as the sensor chip, which was assembled to form a pressure sensor. Finally, experiments were carried out to evaluate the performance.

## 2. Structure Design and Simulation

### 2.1. Structure Design

In this paper, a novel structure featuring four-grooved membrane combined with rood beam was designed for the sensor chip to measure low pressure of less than 1 psi. A 4 inch n-type (100) oriented SOI wafer with 30 μm top silicon, 2 μm buried SiO_2_ layer and 300 μm bottom silicon was chosen as substrate.

**Figure 1:**
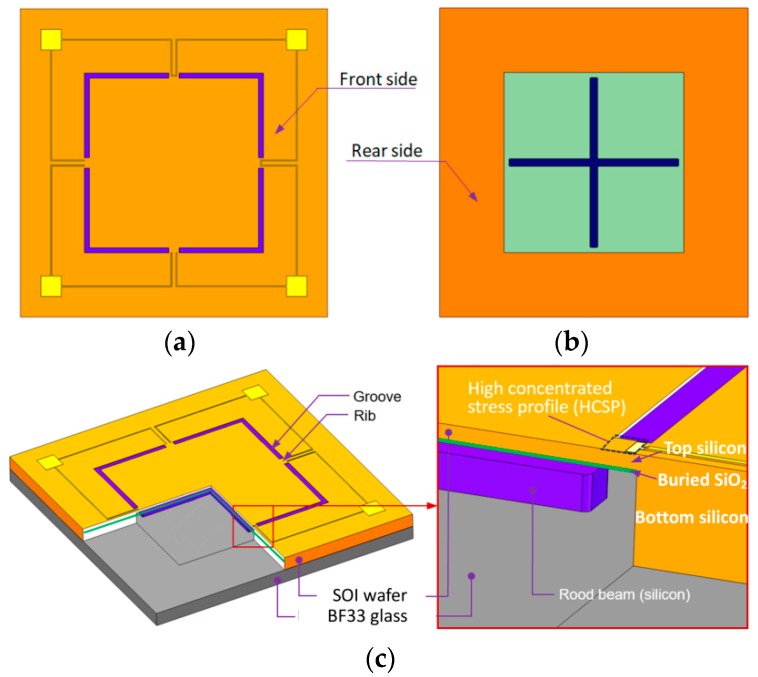
Sketch of the proposed membrane: (**a**) front view; (**b**) rear view; and (**c**) partial section view and detailed structure around the rib place.

On the front side, there are four bending grooves on the membrane surface as shown in Figure 1a. Moreover, four ribs are located between every two grooves which are just on the top of the gap between each beam and membrane edge. Then, a Wheatstone bridge is built up through electric connections with the four piezoresistors on the surface of the rib regions.

On the rear side, a rood beam structure is placed as shown in Figure 1b. The end of each rood beam is not connected with membrane edge but remains at a distance between them, which can be seen clearly in Figure 1c. Meanwhile, the rib width is equal to the groove width, and the rib length is equal to the rood beam width, which not only simplifies the fabrication processes, but facilitates the dimension variables optimization. By incorporating four grooves and a rood beam into the membrane, a high concentrated stress profile (HCSP) was anticipated to maximize the sensitivity. Meanwhile, a locally stiffened membrane (LSM) structure was anticipated to be generated to decrease the deflection of the plate but not reduce the stress around the HCSP. 

### 2.2. Mechanical Simulation

**Figure 2:**
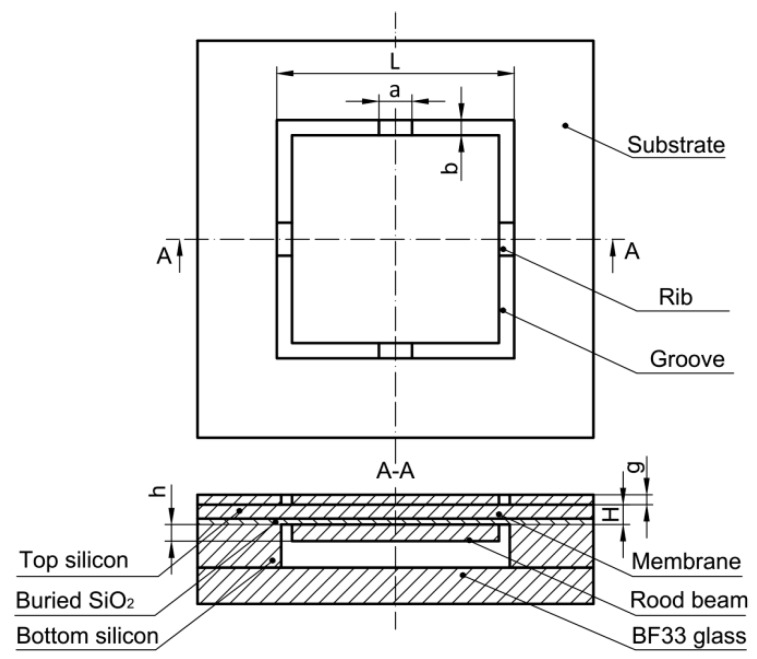
Front view and cross-sectional view for the proposed structure.

To better display the geometry of the structure, the front view and cross-sectional view along A-A of the proposed chip are presented in Figure 2, marked with structural dimension variables. *L* is the membrane length, *H* is the membrane thickness, *b* is the groove width, *g* is the groove depth, and *a* and *h* are rood beam width and thickness, respectively. Meanwhile, the bonding BF33 glass on the rear side can be found obviously from the cross-sectional view. Based on the previous design experiences [7,9,14], the scope for each structural dimension variable is followed by:

$$\left\{ \begin{array}{l} 3000\leq L\leq 4000\\ 20\leq H\leq 40\\ 20\leq h\leq 50\\ 160\leq a\leq 240\\ 40\leq b\leq 120\\ 0\leq g\leq 20 \end{array}\right.$$

**Figure 3:**
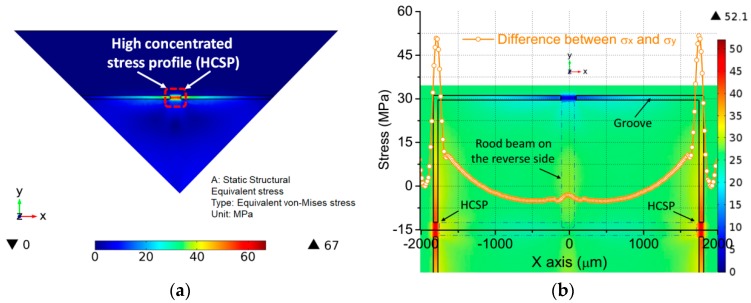
Stress distributions for the proposed membrane: (**a**) high concentrated stress profile on the 1/4 model of the membrane; and (**b**) stress difference variation along x-path.

In the scope for each structural dimension variable, take some fixed size as an example. The stress of the proposed membrane is calculated under 1 psi by non-linear static analysis and model analysis using the commercially-available FEM software COMSOL Multiphysics^®^. Only a quarter of the model is established for the sensitive membrane due to the symmetry as shown in Figure 3a. It can be found that the stress is mainly concentrated at the hinge area which is located at the rib surface, as displayed by the dotted square, which is indicated as HCSP. For P-type [110] oriented piezoresistors, the sensitivity is actually determined by the magnitude of the difference between the transversal stress σ_x_ and the longitudinal stress σ_y_, namely, σ = σ_x_ − σ_y_. It can be seen that the maximum stress along x-path appears at HCSP, but the stress in other regions of membrane is close to zero, as shown in Figure 3b. It illustrates that the strain energy is strictly limited in a narrow area and the energy is not spread easily outside the HCSP. There is a small distance between the rood beam and edge on the back side of the membrane. This gap is used to form a stiffness mutation, which can further enhance the stress concentration. Then, the resistors can experience more stresses and strains, the sensitivity will be improved again.

**Figure 4:**
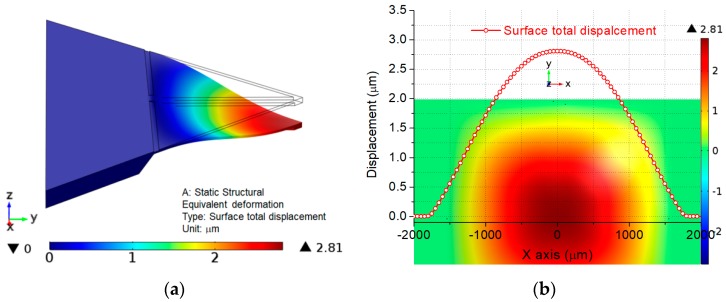
Surface total displacement for the proposed membrane: (**a**) displacement on the 1/4 model of the membrane; and (**b**) displacement variation along x-path.

According to the ratio between membrane deformation and thickness, the deflection theory can be divided into small-deflection theory and large-deflection theory. Small-deflection theory is always chosen to describe the principle of the sensor but large-deflection theory is usually adopted to illustrate the phenomenon of nonlinearity error. Once the deflection exceeds the definite value compared to the thickness of the membrane, the large deflection theory works, and, then, linearity between deflection and pressure will change to nonlinearity [28]. Based on the large deflection curve, the relationship between pressure and deflection is no longer linear when the ratio of (membrane deflection)/(membrane thickness) is larger than 0.2 [29]. It indicates the small-deflection theory only works when the deflection is smaller than 1/5 of the membrane thickness. Thus, the maximum deflection should be less than 1/5 of the membrane thickness, which is beneficial for obtaining a low pressure nonlinearity error. The simulation of surface displacement only displays a 1/4 model of the membrane because of the symmetry. The maximum surface displacement appears at the center of the membrane as shown in Figure 4a. The maximum displacement value reaches 2.8 μm, which is almost 1/10 of the membrane thickness (30 μm), as shown in Figure 4b, and totally satisfies the principle of small-deflection theory.

### 2.3. Geometry Optimization

To determine the geometry of the sensitive membrane, the relationship between the dimension variables and mechanical performance should be discussed. In this section, a combination of FEM simulation and curve fitting method is chosen to deduce the theoretical formulas of the proposed membrane.

Based on the formulas of traditional C-type membrane, the maximum stress and deflection of the membrane are the power functions of each single structural dimension variable [30,31]. In theory, the formulas for the proposed structure should be similar to C-type. Consequently, the functional forms are followed by:

(1) $$\sigma_{max}=Q_{1}\cdot P^{c1}\cdot E^{d1}\cdot L^{i1}\cdot H^{k1}\cdot h^{m1}\cdot a^{n1}\cdot b^{r1}\cdot g^{s1}$$

(2) $$\omega_{max}=Q_{2}\cdot P^{c2}\cdot E^{d2}\cdot L^{i2}\cdot H^{k2}\cdot h^{m2}\cdot a^{n2}\cdot b^{r2}\cdot g^{s2}$$

(3) $$\sigma_{overload}=Q_{3}\cdot E^{d3}\cdot L^{i3}\cdot H^{k3}\cdot h^{m3}\cdot a^{n3}\cdot b^{r3}\cdot g^{s3}$$

where *L*, *H*, *h*, *a*, *b*, and *g* are the structural dimensions as shown in Figure 2; *P* and *E* are the applied pressure and Young’s elastic modulus, respectively; σ*_max_*, ω*_max_* and σ*_overload_* are the maximum von Mises stress, maximum deflection, and maximum von Mises stress under a fixed pressure (100 psi), respectively; and *Q*_1_, *Q*_2_, *Q*_3_, *c*_1_, *c*_2_, *d*_1_, *d*_2_, *d*_3_, *i*_1_, *i*_2_, *i*_3_, *k*_1_, *k*_2_, *k*_3_, *m*_1_, *m*_2_, *m*_3_, *n*_1_, *n*_2_, *n*_3_, *r*_1_, *r*_2_, *r*_3_, *s*_1_, *s*_2_, and *s*_3_ are the curve fitting coefficients. 

To calculate above coefficients, each single dimension variable should be discussed separately. For instance, when membrane length *L* is discussed, other variables have to be assumed as constants. The values of these variables are given initially and arbitrarily in the ranges of actual microfabrication. Thus, Equations (1)–(3) can be simplified to:

(4) $$\sigma_{max}=Q_{1l}\cdot L^{i1}$$

(5) $$\omega_{max}=Q_{2l}\cdot L^{i2}$$

(6) $$\sigma_{overload}=Q_{3l}\cdot L^{i3}$$

where *Q*_1l_, *Q*_2l_ and *Q*_3l_ are the coefficients for the variable *L*, and other parameters have been mentioned. Since the variation of the membrane length *L*, a series of σ*_max_*, ω*_max_* and σ*_overload_* will be obtained by FEM numerical calculation. Then, fitting curves and the coefficients *Q*_1*l*_, *Q*_2*l*_, *Q*_3*l*_, *i*_1_, *i*_2_, and *i*_3_ are obtained by utilizing the software Origin in accordance with simulation results. Then, Equations (4)–(6) are followed by:

(7) $$\sigma_{max}=7.8396\times 10^{-8}L^{2.51041}$$

(8) $$\omega_{max}=4.5474\times 10^{-15}L^{4.15924}$$

(9) $$\sigma_{overload}=6.5390\times 10^{-6}L^{2.5313}$$

Using the same approach, the fitting equations and curves related to other membrane dimensions can also be deduced. Then, equations about membrane thickness *H* are derived as:

(10) $$\sigma_{max}=7.2646\times 10^{4}H^{-2.0675}$$

(11) $$\omega_{max}=8.0452\times 10^{3}H^{-2.34447}$$

(12) $$\sigma_{overload}=7.0142\times 10^{6}H^{-2.05714}\;\;$$

In addition, the equations related to groove width *b* are shown as:

(13) $$\sigma_{max}=131.9712b^{-0.16719}$$

(14) $$\omega_{max}=1.2189b^{0.20466}$$

(15) $$\sigma_{overload}=1.2834\times 10^{4}b^{-0.016643}\approx 1.2834\times 10^{4}$$

According to Equation (15), it can be concluded that groove width *b* has little influence on the σ*_overload_*. Moreover, the equations with respect to groove depth *g* are listed as:

(16) $$\sigma_{max}=32.2032g^{0.32476}$$

(17) $$\omega_{max}=2.1207\times 10^{3}g^{0.12861}$$

(18) $$\sigma_{overload}=3.2382\times 10^{3}g^{0.32139}$$

For rood beam width *a*, the relative equations are followed by:

(19) $$\sigma_{max}=218.3372a^{-0.22859}$$

(20) $$\omega_{max}=20.6422a^{-0.37743}$$

(21) $$\sigma_{overload}=2.4904\times 10^{4}a^{-0.25478}$$

In the same way, the equations regarding rood beam thickness *h* are:

(22) $$\sigma_{max}=116.5183h^{-0.17377}$$

(23) $$\omega_{max}=17.7924h^{-0.52592}$$

(24) $$\sigma_{overload}=1.1682\times 10^{4}h^{-0.017581}\approx 1.1682\times 10^{4}$$

Equation (24) reveals that there is tiny impact of the rood beam thickness *h* on the σ*_overload_*. The equations about pressure *P* are followed by:

(25) $$\sigma_{max}=9.4421\times 10^{-3}P^{1.00062}\approx 9.4421\times 10^{-3}P$$

(26) $$\omega_{max}=3.7268\times 10^{-4}\times P^{0.99547}\approx 3.7268\times 10^{-4}P\;\;\;$$

From Equations (25) and (26), the stress and deflection of the membrane show linear relationships with the pressure *P*. Finally, the equations related to Young’s elastic modulus *E* are expressed as follows:

(27) $$\sigma_{max}=9.3215\times 10^{6}E^{-0.0025789}\approx 9.3215\times 10^{6}$$

(28) $$\omega_{max}=1.2453\times 10^{5}E^{-0.98451}\approx 1.2453\times 10^{5}E^{-1}$$

(29) $$\sigma_{overload}=6.895\times 10^{9}E^{-0.0421}\approx 6.895\times 10^{9}$$

Equations (27)–(29) not only illustrate that the Young’s elastic modulus *E* has almost no effect on σ*_max_* and σ*_overload_*, but also reveal the inverse proportional relationship between *E* and maximum deflection ω*_max_*. By combining all the equations related to each single variable, the particular equations of Equations (1)–(3) can be determined. Simultaneously, to calculate the constant coefficients *Q*_1_, *Q*_2_, and *Q*_3_, a set of particular solutions are introduced, and thus the relative main equations can be derived as follows:

(30) $$\sigma_{max}=7.36\times 10^{-2}\frac{PL^{2.51041}g^{0.32476}}{H^{2.0675}h^{0.17377}a^{0.22859}b^{0.16719}}$$

(31) $$\omega_{max}=5.64\times 10^{-3}\frac{PL^{4.15924}b^{0.20466}g^{0.12861}}{EH^{2.3447}h^{0.52592}a^{0.37743}}$$

(32) $$\sigma_{overload}=2.45\times 10^{3}\frac{L^{2.5313}g^{0.32139}}{H^{2.05714}a^{0.25478}}$$

From Equations (30)–(32), it can be found that with the increase of the membrane length *L*, the maximum stress and deflection, both, experience a rise, namely, improve sensitivity, but worsen linearity. Meanwhile, the impacts for the membrane thickness *H*, rood beam thickness *h*, rood beam width *a* and groove depth *g* on the sensitivity and linearity are same except groove width *b*. It is the only variable which is inversely proportional to the stress and directly proportional to the deflection. It means that the sensitivity and linearity can be improved synchronously when the groove width is chosen appropriately. Besides, groove width *b* and rood beam thickness *h* have nearly no influences on σ*_overload_*. 

**Figure 5:**
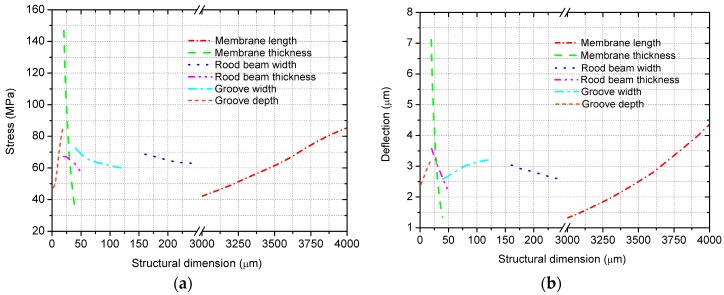
Mechanical performance variations versus the structural dimension variables: (**a**) stress variation; and (**b**) deflection variation.

The impacts of structural dimension variables on the stress and deflection variations are presented in Figure 5a,b. To determine the optimal solution of the nonlinear optimization problem, MATLAB is used. By the fine adjustment of the MATLAB calculated results, a series of structural dimensions are finally determined based on comprehensively regarding the influences on stress and deflection of the membrane as listed in Table 1.

## 3. Fabrication and Measurements

**Figure 6:**
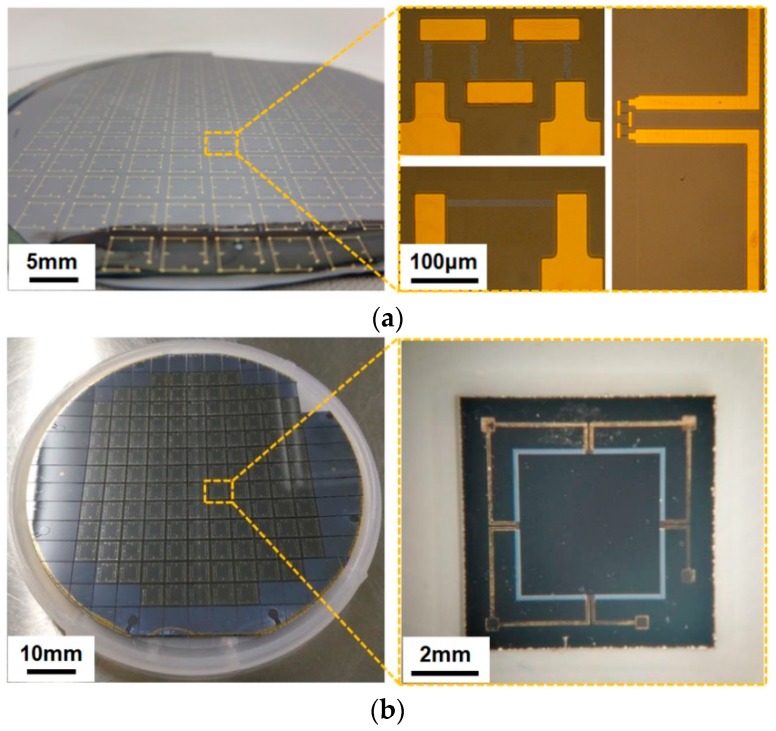
Photographs of the proposed sensor chip: (**a**) front side of the wafer after metallization and its two different pattern piezoresistors and (**b**) photographs of the sensor chip after laser cutting.

A 4-inch (100 ± 0.5 mm) n-type SOI wafer oriented in the (100) direction with a resistivity of 10 Ω·cm was chosen as the substrate of the sensor chip. It is 330 ± 5 μm thickness and double sides polished with a total thickness variation of less than 5 μm. The specific fabrication process of the proposed sensor chip is summarized as follows. The first step is to grow SiO_2_ protective layers on both sides of the wafer by thermal oxidation. A 300 ± 20 nm SiO_2_ deposition layer is formed using wet oxidation at 1000 °C. Then, lithography is performed on the front side of silicon oxide to pattern the piezoresistors. The front side of wafer is implanted with boron ions (dose 4.86 × 10^14^ atoms/cm^2^, energy 70 keV) and subsequently annealed at 1000 °C for 30 min in N_2_ ambient for dopant activation. P-type resistors (210 ± 10 Ω/Sq.) and heavy doping of contact regions are formed in the doped region. After that, the low pressure chemical vapor deposition (LPCVD) is adopted to grow the passivation layers of Si_3_N_4_ to protect the piezoresistors. Subsequently, the reactive ion etching (RIE) process is used to etch the ohm contact regions between the piezoresistors and the metal lead. Next, Cr/Au (50 nm/200 nm) coatings are deposited and patterned for the purpose of connecting of resistors and forming of bonding pads as presented in Figure 6a. In the following, RIE is applied to create four grooves on the front side, and DRIE is used to form the cavity and rood beam on the rear side. Moreover, it is noteworthy that the rear side etching is processed in two steps. The first step is to etch rood beam patterning by one mask (etching depth was 35 μm). The second step is to form the cavity and rood beam structure simultaneously by another mask. To obtain an absolute pressure reference chamber, the bottom side of the wafer is attached to the BF33 glass by anodic bonding process under vacuum condition (5 × 10^−6^ Bar) at 360 °C with an electric field of 600 V (20 min). The photographs of the sensor chip after laser cutting are shown in Figure 6b. 

**Figure 7:**
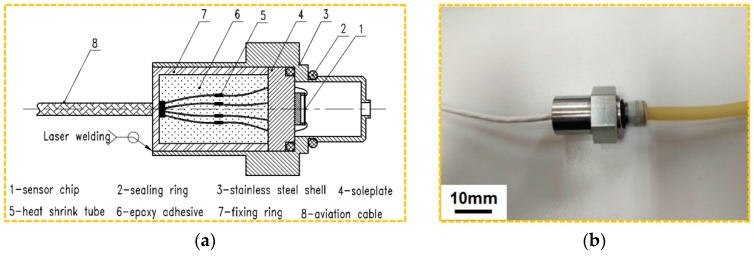
Schematic diagram of the assembling: (**a**) interconnecting structure; and (**b**) assembled sensor.

The pressure sensor is assembled with the fabricated sensor chip as shown in Figure 7a. The sensor is mainly constituted by chip, shell, soleplate, aviation cable, etc. The metal material of the external structure is 304 stainless steel which is beneficial to improve the corrosion resistance. To increase the sealing and insulation characteristics of the sensor, the epoxy adhesive (60% ethoxyline resin, 30% quartz powder, 10% dioctyl phthalate) is filled in the shell and solidified for about 24 h at room temperature (25 °C). An aviation cable is used to connect the sensor and the plug, which can resist high temperatures (>200 °C). To protect the welding spot, the fluororubber heat shrink tube is covered at the connection between every two wires. Between the stainless steel shell and fixing ring, laser welding is chosen for connection, which can improve the long term stability and application of the sensor. As a result, the assembled sensor is presented in Figure 7b. 

**Figure 8:**
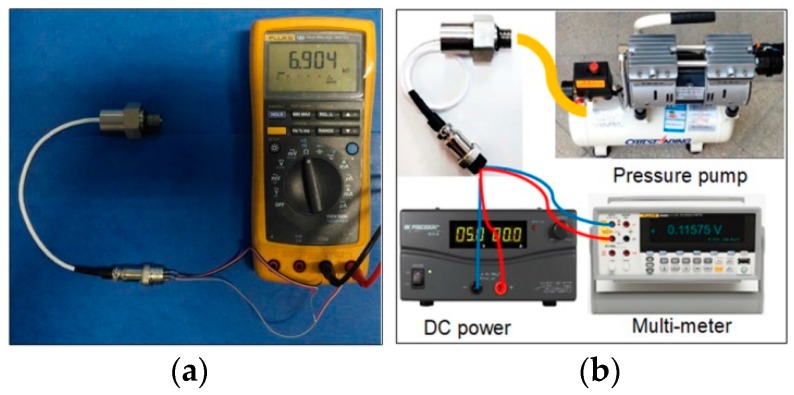

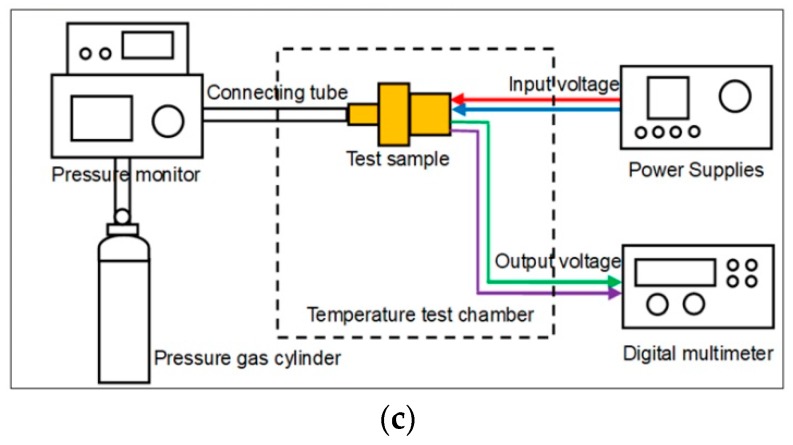
Experimental schematic diagrams for the proposed sensor: (**a**) test diagram for bridge resistance; (**b**) test diagram for output voltage at room temperature (25 °C); and (**c**) test diagram at different temperatures.

The performances of the sensor are measured and the experimental schematic diagrams for testing are shown in Figure 8. The bridge resistance is measured by a digital multimeter (VC890D). The pressure is from a pressure pump and the pressure value can be adjusted by controlling switch. To ensure the airtightness of the measurement, the joint between the sensor port and connecting tube should be twined several laps using the rubber belt. A constant voltage of 5 V is provided to the Wheatstone bridge of the sensor using a High Current Switching DC Power Supplier (Model BK1694). The output of the sensor at different pressure loads is read by a digital multimeter (FLUKE 8845A). The output voltages at different temperatures are measured in a high and low temperature test chamber (GDC4010) with the working range of −20 to 150 °C. To test the characteristics of the sensor at different temperatures, nine temperature points are tested in the range of −20 to 150 °C: −20 °C, −10 °C, 0 °C, 25 °C, 50 °C, 75 °C, 100 °C, 125 °C and 150 °C. The purpose is to check the temperature characteristic of the device. In the process of high temperature measurement, the test step change of pressure is set as 0.1 psi, namely, a total number of eleven test points is selected. Based on the pressure sensor calibration procedures, the pressure signals increase from low to high until 1 psi, and then decrease from 1 psi to 0. The above process is called “one trip”. For each temperature point, three trips are carried out for a precise measurement. For each test point in each trip, the output data will be not read until the pressure is stable. Finally, the various indicators of the sensor are calculated. 

## 4. Results and Discussion

After assembling the sensor, the first step is to connect the plug and socket to measure the bridge resistance. The resistances of three samples are listed in Table 2. Test results show that the consistency of the resistance is not high enough. This is mainly because the connecting regions between piezoresistors and Cr-Au wires have some different degrees of the defects. Besides, most of the measured resistances are smaller than the designed value 6.7 kΩ. This is due to the errors involved in ion implantation and sputtering processes. The resistance of Device 2 is the closest to the design value, so it is chosen as the sample to be tested at different conditions.

**Figure 9:**
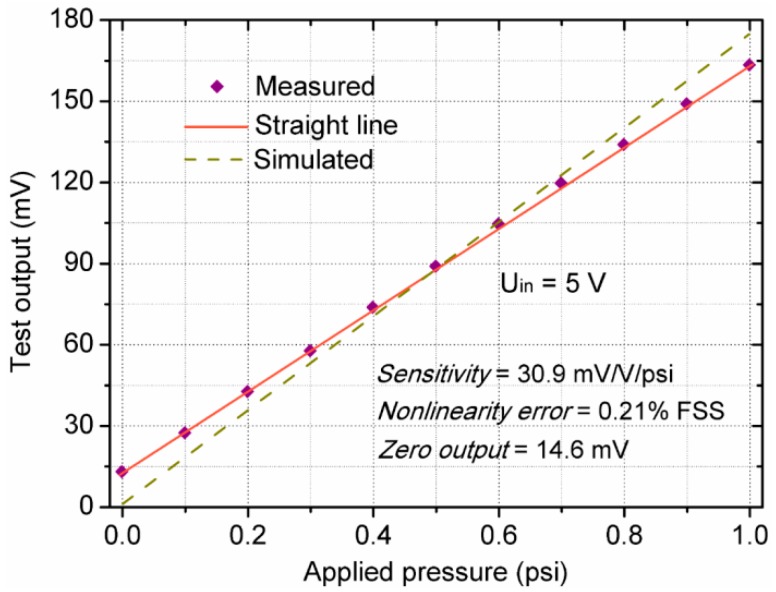
Output voltage of the sensor versus applied pressure.

The performance of one pressure sensor is usually evaluated by the static characteristics of technical indicators including full scale output, sensitivity, pressure nonlinearity error, repeatability, hysteresis, zero output, accuracy, etc. [32]. The measured output voltage and pressure nonlinearity error are shown in Figure 9. In this paper, the accuracy is calculated using root of the sum squared to combine nonlinearity, repeatability, and hysteresis into a total accuracy percentage. Finally, the detailed technical data of the sensor at room temperature (25 °C) are listed in Table 3. The results illustrate that the proposed sensor achieves a high sensitivity of 30.9 mV/V/psi and a low nonlinearity error of 0.21% FSS (full scale span). Meanwhile, there is a deviation of 8.4% between the simulated and experimental results. It is illustrated that the estimated data are similar to the actual data, which proves the validity of the simulation and optimization methods.

**Figure 10:**
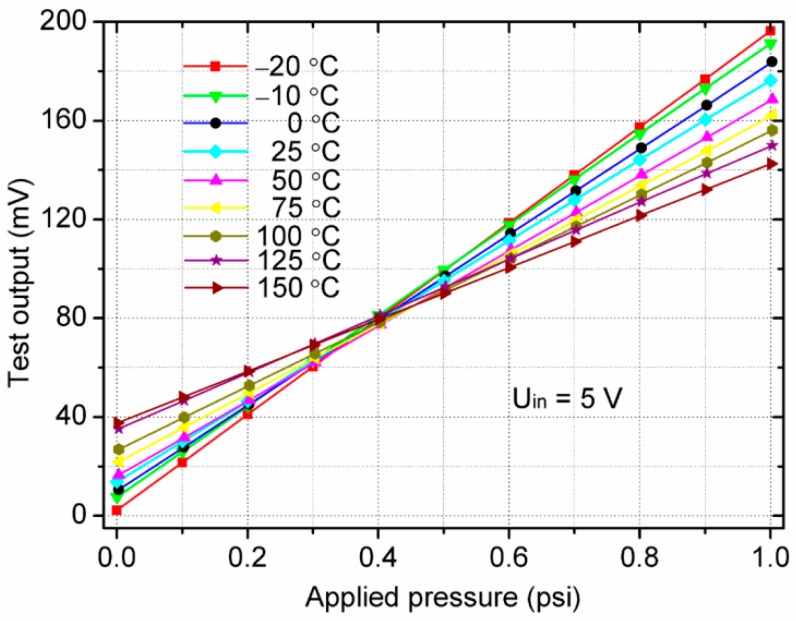
Sensor input–output curves at different temperatures.

The sensitivity and zero output analyses are the study of change in output voltage with respect to the applied pressure at different temperatures. Figure 10 illustrates the relationship between the output of the sensor and the standard pressure under different temperatures. It can be observed that the lower the temperature is, the larger the output voltage is. It is also observed that the output increases linearly with the applied pressure. However, the output curves of the sensor at different temperatures are not coincident and there are also differences in the zero output voltage as well as the sensitivity, which demonstrates that the proposed sensor has a temperature drift. It is consistent with the conclusion of References [33,34].

**Figure 11:**
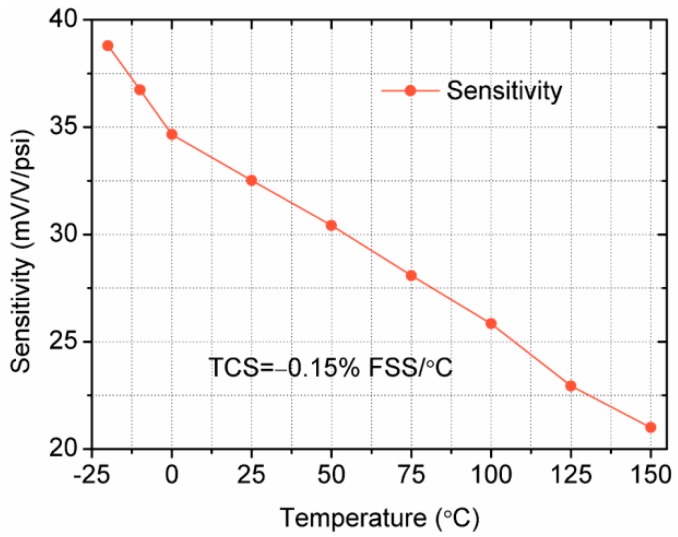
Curve of the sensitivity variation at different temperatures under 1 psi.

To be more intuitional, Figure 11 displays the sensor’s sensitivity changes with the temperature increasing when the test pressure is 1 psi. The results show that the sensitivity reduces almost by 50% when the temperature increases from −25 °C to 150 °C. This is mainly because the piezoresistive coefficient decreases when the temperature rises. However, the sensitivity is still as high as 21.2 mV/V/psi at 150 °C. The temperature coefficient of sensitivity (TCS) is calculated using the full output at 150 °C and reference temperature 25 °C, where the TCS of the sensor is −0.15% FSS/°C, indicating that the sensitivity of the sensor has a negative temperature coefficient. 

**Figure 12:**
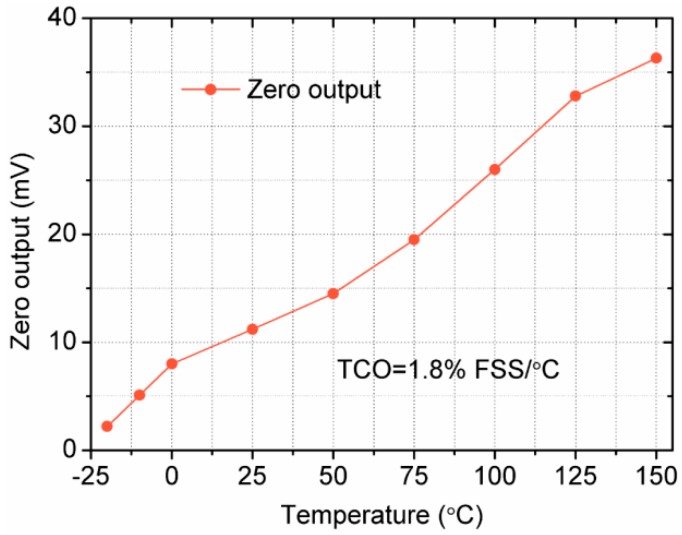
Zero output of the sensor at different temperatures.

The zero output voltage increases substantially when the temperature is rising, as shown in Figure 12. The zero output comes from two aspects. The first reason is due to some residual stress on the membrane. The second reason is because of the non-uniformity in the doping of the four piezoresistors. The high value of zero output at high temperature may be attributed to the difference in the shapes of piezoresistors experiencing longitudinal and transverse stresses [35]. It is found that the zero output increases 15.5 times when the temperature is changing from −20 °C to 150 °C, which indicates the proposed sensor possesses an obvious temperature-sensitive character. By calculation, the temperature coefficient of voltage offset (TCO) of the sensor is 1.8% FSS/°C. 

Generally, two methods are usually adopted to reduce offset voltage. On the one hand, the piezoresistive coefficients are the function of the temperature and doping concentration, namely, π*(N,T)* = π_0_ × *P(N,T)*. According to the piezoresistance factor influenced by the temperature and impurity concentration for monocrystalline silicon, a lower doping concentration is commonly needed [36]. On the other hand, the offset voltage can also be decreased by incorporating compensation circuits and signal-conditioning circuits either by using them on the silicon die itself or by employing hybrid components/signal conditioning ICs/resistor.

**Figure 13:**
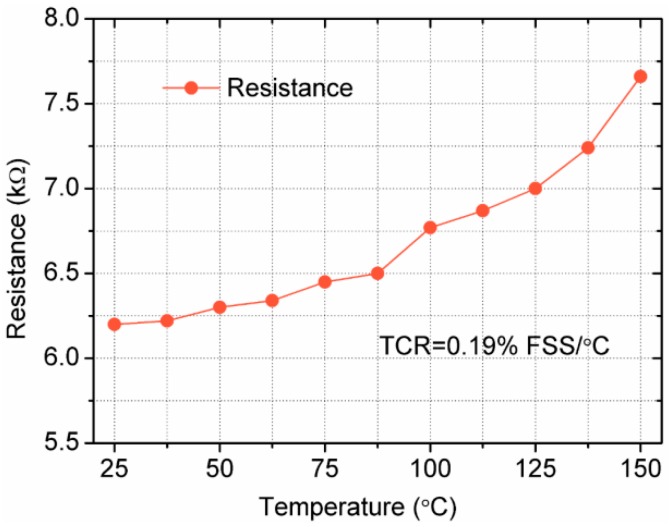
Curve of the resistance variation at different temperatures.

With the temperature increases, the resistance experiences a rise until reaching the largest value at 150 °C, as shown in Figure 13. Commonly, one requirement of analog electronics is the positive temperature coefficient of the sensitivity over the entire temperature range. Thus, the positive temperature coefficient of resistivity (TCR) has to predominate the negative temperature coefficient of the gauge factor [37]. Based on the resistance variation, TCR is calculated as 0.19% FSS/°C. 

The proposed sensor demonstrates that it does have the ability to alleviate the contradiction between sensitivity and linearity to realize the micro measurement with high accuracy, as shown in Table 4. Compared with the four other reported sensors, the sensitivity of the proposed sensor is the second largest, which is more than three times larger than that reported in [38]. At the same time, the pressure nonlinearity for the sensor is intermediate among the five types of sensors, unlike [39] where the small pressure nonlinearity is obtained but its sensitivity is too low. Thus, it can be concluded that the proposed sensor achieves a high sensitivity and a low pressure nonlinearity error when compared with the reported sensors. The proposed sensor not only achieves a good performance by nonlinear optimization of the structure, but also obtains a good output characteristic at high temperature using SOI as the substrate. In other words, the sensor in this work is the only candidate that can work in high temperature, when compared to the four other reported devices.

Based on the measurement at different temperatures, the temperature characteristic for the proposed sensor is illustrated. The sensitivity is 21.2 mV/V/psi, the nonlinearity is 0.25% FSS, the repeatability is 0.19% FSS, the hysteresis is 0.13% FSS, and so the accuracy is up to 0.34% FSS. Obviously, the sensor also satisfies the linearity at high temperature. As the temperature is rising, the characteristic has some degradation, but, overall, the change in accuracy is not obvious. The sensor maintains fine characteristic at high temperature. The contrast in accuracy between the proposed sensor and several previously reported sensors is displayed in Table 5. Compared with the other reported sensors, the proposed sensor achieves the best linearity. Moreover, the accuracy for the sensor is intermediate among the four sensors. Therefore, it can be concluded that the proposed sensor achieves a high accuracy at high temperature when it is compared with several reported sensors.

## 5. Conclusions

In this study, a novel structural piezoresistive pressure sensor was developed by introducing a rood beam into a four-grooved membrane. The proposed structure provided a solution for enhancing sensitivity and linearity simultaneously. To testify the feasibility of the scheme, the proposed model was simulated, optimized, fabricated and measured. Test results illustrated the effectiveness of incorporating grooves and a rood beam to improve accuracy and alleviate the trade-off between sensitivity and linearity. Besides, the fabricated pressure sensor exhibited high linearity and accuracy at high temperature. Thus, in terms of micro size, accuracy and high temperature characteristic, the proposed structure was a good candidate for measuring low pressure in the range of 0–1 psi under 150 °C.

## Figures and Tables

**Table 1:** Optimized dimensions of the proposed membrane.

Parameters	*L*	*H*	*h*	*a*	*b*	*g*
Dimension (μm)	3600	30	35	200	60	10

**Table 2:** Test results of the Wheatstone bridge resistance.

Sample	*R*_1_ (kΩ)	*R*_2_ (kΩ)	*R*_3_ (kΩ)	*R*_4_ (kΩ)
Device 1	5.123	4.866	5.469	4.773
Device 2	6.678	6.125	6.904	6.439
Device 3	5.562	5.245	5.417	5.183

**Table 3:** Technical data of the sensor at the room temperature.

Parameter	Value	Parameter	Value
Input voltage (V)	5	Repeatability (%FSS)	0.17
Resistance (kΩ)	6.7	Hysteresis (%FSS)	0.12
Zero output (mV)	14.6	Accuracy (%FSS)	0.30
Full range output (mV)	169.1	TCS (%FSS/°C)	−0.15
Sensitivity (mV/V/psi)	30.9	TCO (%FSS/°C)	1.8
Pressure nonlinearity (% FSS)	0.21	TCR (%FSS/°C)	0.19

**Table 4:** Comparison in performance with other pressure sensors at room temperature.

Sensor	Sensitivity (mV/V/psi)	Pressure Nonlinearity (% FSS)	Accuracy (% FSS)	Full Range Pressure (kPa)
Proposed sensor	30.9	0.21	0.30	6.894
Sensor in [38]	10.1	0.19	0.24	10
Sensor in [39]	12.1	0.05	0.68	10
Sensor in [23]	25.4	0.36	-	5
Sensor in [40]	32.1	0.25	0.34	5

**Table 5:** Comparison in accuracy with other pressure sensors at high temperature.

Sensor	Pressure Nonlinearity (% FSS)	Repeatability (% FSS)	Hysteresis (% FSS)	Accuracy (% FSS)	Temperature (°C)
Proposed sensor	0.25	0.19	0.14	0.34	150
Sensor in [41,42]	0.33	0.22	0.13	0.72	60
Sensor in [43]	0.26	0.11	0.11	0.3	200
Sensor in [6]	0.3	0.16	0.14	0.48	200
